# Nicotinamide Mononucleotide Enhances Boar Sperm Quality via Maintaining Mitochondrial Function During Liquid Storage

**DOI:** 10.3390/ani15233383

**Published:** 2025-11-22

**Authors:** Yongjin Liu, Hongyan Zhang, Qingzhe Meng, Lingjiang Min, Min Zhang, Adedeji O. Adetunji, Wenjing Li, Zhendong Zhu

**Affiliations:** 1 College of Animal Science and Technology, Qingdao Agricultural University, Qingdao 266109, China; lyjxl76198@163.com (Y.L.); 20232209018@stu.qau.edu.cn (H.Z.); 20242109037@stu.qau.edu.cn (Q.M.); mljlab405@qau.edu.cn (L.M.); 2 Protection of Animal Genetic Resources and Biological Breeding Engineering Research Center of Shandong Province, Jinan 250300, China; 18866617369@163.com; 3 Department of Agriculture, University of Arkansas at Pine Bluff, Pine Bluff, AR 71601, USA; adetunjiadedeji.aa@gmail.com; 4 Key Laboratory of Swine Breeding in South China, Ministry of Agriculture and Rural Affairs, Aonong Group, Zhangzhou 363000, China

**Keywords:** boar, nicotinamide mononucleotide, mitochondrial function, sperm quality, capacitation

## Abstract

Nicotinamide mononucleotide (NMN), a key precursor of nicotinamide adenine dinucleotide (NAD^+^), plays a central role in cellular energy metabolism. This study investigated the effect of NMN supplementation on boar sperm quality during liquid storage. Semen samples diluted with Modena extender containing 0 to 80 μM NMN were stored at 17 °C for 7 days. Results demonstrate that supplementation with 20 μM NMN significantly improved sperm motility, acrosome integrity, and mitochondrial activity compared with the control group, accompanied by markedly elevated intracellular NAD^+^ and ATP level (*p* < 0.05). Also, Western blot analysis confirmed the presence of nicotinamide mononucleotide adenylyltransferase 3 (NMNAT3) in boar sperm. Furthermore, sperm treated with 20 μM NMN exhibited a higher level of protein tyrosine phosphorylation and an increased capacitation rate following storage. Tissue explant assays further revealed a significant increase in the number of sperm attached to oviductal epithelial fragments, indicating enhanced sperm–oviduct interactions. The present findings demonstrate that 20 μM NMN supplementation effectively preserves the metabolic activity and functional competence of boar sperm during liquid storage. It provides a promising strategy for improving boar semen preservation.

## Simple Summary

Nicotinamide mononucleotide (NMN) is a key precursor of nicotinamide adenine dinucleotide (NAD^+^) and plays a vital role in cellular energy metabolism. This study investigated the effects of NMN supplementation on boar sperm quality during liquid storage. At a concentration of 20 μM NMN, sperm motility and mitochondrial activity were maintained for up to 7 days at 17 °C. These results indicate that NMN supports sperm energy metabolism and function even at low doses, offering a cost-effective strategy to enhance semen preservation in the swine breeding industry.

## 1. Introduction

In swine artificial insemination (AI), sperm motility plays a critical role in achieving successful fertilization and increasing litter size [1]. Sperm motility depends on ATP, which is mainly generated through glycolysis and mitochondrial oxidative phosphorylation [2]. Mitochondrial activity is positively associated with sperm motility and survival [3,4]. Since mitochondrial metabolism requires cofactors such as nicotinamide adenine dinucleotide (NAD^+^) [5], maintaining or enhancing NAD^+^ availability may optimize mitochondrial efficiency, thereby sustaining sperm motility and energy supply [6].

Mitochondria are densely clustered in the sperm midpiece, where they serve as the primary source of continuous ATP production required for sperm motility [7]. The functional integrity of these organelles directly determines sperm motility, as demonstrated by studies linking mitochondrial membrane potential (MMP) with motility status [8]. Higher MMP correlates with vigorous movement, while its decline precipitates motility dysfunction, DNA damage, and functional impairment [9,10]. The activities of mitochondrial electron transport chain enzymes are crucial in regulating ATP synthesis rates [11]. This central role is further underscored by studies that show FCCP (carbonyl cyanide p-trifluoromethoxy phenylhydrazone) inhibits mitochondrial function, rapidly depleting ATP and halting motility [12]. However, mitochondrial ATP generation inherently produces reactive oxygen species (ROS). Excessive ROS can disrupt sperm membranes and DNA, thereby reducing motility [13,14,15]. Taken together, effective mitochondrial energy metabolism is vital for sperm movement, and its efficiency is closely associated with cofactors such as NAD^+^ that facilitate ATP production.

NAD^+^ is central to mitochondrial metabolism, a pivotal coenzyme facilitating electron transfer in the TCA cycle and oxidative phosphorylation [16]. Adequate NAD^+^ level is indispensable for proper mitochondrial function [17,18]. As a direct NAD^+^ precursor, Nicotinamide mononucleotide (NMN) effectively elevates NAD^+^ concentrations and enhances mitochondrial performance across mammalian cell types [19]. Previous studies have shown that NMN supplementation activates NAD^+^-dependent enzymes such as Sirtuin 3 (SIRT3), thereby enhancing mitochondrial oxidative phosphorylation and improving sperm energy metabolism [20,21].

Therefore, this study aimed to investigate how exogenous nicotinamide mononucleotide (NMN) supplementation affects boar sperm motility and mitochondrial function during liquid storage. We hypothesized that NMN enhances sperm energy metabolism by increasing intracellular nicotinamide adenine dinucleotide (NAD^+^) level, thereby sustaining mitochondrial activity and maintaining sperm function throughout storage. By further elucidating the metabolic and mitochondrial mechanisms underlying NMN action during liquid storage, this study enhances current understanding of NMN’s role in maintaining sperm quality and provides valuable evidence for improving semen preservation.

## 2. Materials and Methods

### 2.1. Media Preparation

As described by Zhu et al. [22], the Modena solution was used as an extender to dilute boar semen. The capacitation medium was prepared based on previously reported formulations by Ded et al. [23], with the following composition: 11.3 mM NaCl, 0.3 mM KCl, 1 mM CaCl_2_, 2 mM Tris, 1.1 mM glucose, 0.5 mM sodium pyruvate, and 0.1% BSA.

### 2.2. Animals, Semen Collection and Processing

Nine healthy and fertile Duroc boars provided by Jimo Runmin Co., Ltd. (Qingdao, China) were used in this study. Semen was collected from each boar every five days using gloved techniques, with the sperm-rich fraction filtered through double-layered sterile gauze. Boar semen was immediately diluted with Moderna solution (1:1, *v*:*v*) post-collection. The diluted samples were promptly transported to the laboratory within 30 min. Only ejaculates meeting the criteria that have a motility of over 90% and an abnormal sperm ratio of less than 15% were used in this study. To reduce individual variability, the ejaculates were pooled and divided into six groups to dilute with Modena solution containing varying doses of NMN (0, 5, 10, 20, 40, and 80 μM) at a concentration of 3 × 10^7^ sperm/mL. Subsequently, samples underwent slow cooling to 17 °C and were stored in a thermostatic chamber at 17 °C until analysis.

### 2.3. Sperm Motility

Sperm motility of preserved samples was assessed at 1, 3, 5, and 7 days using a Computer-Assisted Sperm Analysis system (CASA, CEROS II; Hamilton Thorne Inc., Beverly, MA, USA). Following 20 min of incubation at 37 °C, 10 µL aliquots were loaded onto pre-warmed slides, with motility parameters evaluated across three randomized microscopic fields.

### 2.4. Evaluation of Sperm Viability and Acrosome Integrity

As described by Wang et al. [24] in a previous study, a dual-staining protocol using Fluorescein Isothiocyanate-conjugated Peanut Agglutinin (FITC-PNA) and Propidium Iodide (PI) was applied to evaluate sperm viability and acrosomal status. 1 mL aliquot of diluted semen was supplemented with 0.6 μL FITC-PNA and 0.54 μL PI, followed by gentle vortex mixing and incubated in the dark at 37 °C for 5 min. Flow cytometric analysis (BeamCyte, Changzhou, China) was performed on 20,000 events per sample. Sperm negative for FITC-PNA staining were considered acrosome-intact, while PI-negative sperm were considered viable. The percentage of viable sperm with intact acrosomes (FITC-PNA^−^/PI^−^) was calculated. All analyses were performed in triplicate (*n* = 3).

### 2.5. Evaluation of Mitochondrial Membrane Potential

As described by Wang et al. [24], the mitochondrial membrane potential (MMP) in sperm was assessed using the JC-1 Assay Kit (C2006, Beyotime Biotechnology, Shanghai, China). For the positive control group, semen was pretreated with 10 μM carbonyl cyanide 3-chlorophenylhydrazone (CCCP) and incubated for 20 min. Subsequently, both the treated and positive control groups were stained by adding 400 μL of JC-1 working solution to the sperm suspensions, followed by incubation at 37 °C for 20 min. After incubation, samples were washed twice with JC-1 buffer, resuspended, and analyzed using a flow cytometer (BeamCyte, Changzhou, China), with 20,000 events recorded per sample. All experiments were performed in triplicate (*n* = 3).

### 2.6. Measure of Sperm ATP Level

According to the method reported by Wang et al. [25], the ATP level of sperm was quantified using an ATP Assay Kit (Nanjing Jiancheng Bioengineering Institute, Nanjing, China) following the manufacturer’s protocol. Sperm samples were first homogenized in an ice-water bath. A portion of the homogenate was taken for protein concentration determination. The suspension was heated in a boiling water bath for 10 min, then vortexed for 1 min. Subsequently, 30 μL of the lysate was incubated with the working solution. After incubation, a precipitating reagent was added and the mixture was centrifuged at 1500× *g* for 5 min to obtain the supernatant. The supernatant, chromogenic reagent, and stop solution were sequentially added to a 96-well plate. Absorbance was measured at 636 nm using a microplate reader. All experiments were performed in triplicate (*n* = 3).

### 2.7. Evaluation of NAD^+^ and NADH Content

NAD^+^ extraction was performed using the Coenzyme I NAD (H) content test kit (Nanjing Jiancheng Bioengineering Institute, Nanjing, China) as previously reported by Wang et al. [25]. Pelleted sperm were resuspended in an acidic extraction buffer at a concentration of 5 × 10^8^ sperm/mL and sonicated on ice at 20% amplitude (2 s on/1 s off for 1 min). The samples were then boiled for 5 min, cooled on ice, and centrifuged at 10,000× *g*, 4 °C for 10 min. The supernatant was neutralized with an equal volume of alkaline buffer, re-centrifuged under the same conditions, and the final supernatant was stored on ice. According to the manufacturer’s instructions, the reagents were added to the samples and transferred to a 96-well plate, where the absorbance was measured at 570 nm using a microplate reader to determine the NAD^+^ content.

### 2.8. Western Blotting

Sperm lysates were prepared by sonicating samples in RIPA (Radioimmunoprecipitation Assay) buffer supplemented with protease inhibitors. After lysis, the mixtures were centrifuged at 12,000× *g* for 15 min, and the supernatants were collected and mixed with SDS loading buffer before boiling for 10 min. Proteins were separated on 10% SDS-PAGE gels and transferred onto PVDF membranes (Merck Millipore, Darmstadt, Germany). Membranes were blocked with 5% BSA in TBST (Tris-buffered saline with Tween-20) and incubated with the following primary antibodies: anti-Phosphotyrosine (Abcam, AB179530, Shanghai, China) and anti-NMNAT3 (Abcam, ab230839; 1:1000 dilution). After primary antibody incubation, membranes were incubated with HRP-conjugated secondary antibody (A0208, Beyotime, Shanghai, China) for 1 h. Protein signals were visualized using a chemiluminescent imaging system, and band intensities were quantified using ImageJ software (Version 1.54m, NIH, Bethesda, MD, USA) and normalized to α-tubulin. All experiments were performed in triplicate (*n* = 3).

### 2.9. Thermo-Resistance Test

After 7 days of preservation, the semen samples underwent a thermo-resistance test (TRT) following a modified procedure according to Schulze et al. [26]. The samples were incubated at 37 °C for a duration of up to 300 min. Sperm motility parameters were assessed every hour using a CASA system to track the dynamic changes in motility over time.

### 2.10. Capacitation and Acrosome Reaction Assessment

Sperm samples were centrifuged at 600× *g* for 10 min, the supernatant was discarded, and an equal volume of capacitation medium was added. The samples were then incubated at 37 °C in a humidified incubator with 5% CO_2_ for 4 h to induce capacitation. Sperm capacitation status was assessed by chlortetracycline (CTC) fluorescence assay according to Fraser et al. [27]. Briefly, 100 μL of 7-day-preserved sperm after capacitation treatment was centrifuged (140× *g*, 10 min) and resuspended in 50 μL PBS. The sperm samples were stained with 100 μL CTC solution at 37 °C for 20 min, fixed with 5 μL 12.5% glutaraldehyde in 20 mM Tris-HCl, and examined using a fluorescence microscope (ZEISS DM200LED, Oberkochen, Germany).

### 2.11. Assessment of Sperm-Tissue Fragment Binding Capacity

Following the methodology described in a previous study [28], the relative fertilization potential was evaluated by assessing sperm binding capacity to fallopian tube tissue fragments. Fallopian tube tissue was collected from healthy sows, and tissue fragments were prepared from the isthmus region and stored in Tyrode’s medium. Only tissue fragments with intact ciliated epithelium were selected for the experiment. Sperm were incubated after 30 min of Hoechst 33342 staining. Three tissue fragments per sow were placed in pre-warmed Tait medium (5% CO_2_, 38 °C, 100% humidity) within a 24-well plate and incubated with 2 × 10^5^ sperm for 45 min. After gentle washing to remove unbound cells, tissue sections were placed on slides and covered with coverslips. To minimize edge artifacts, the outermost region of the tissue section was excluded from analysis. Three random fields of view were selected from the remaining area, imaged at different focal planes, and images were synthesized for quantification. Combination density was defined as the ratio of bound sperm count to tissue section surface area.

The average sperm bound per mm^2^ was defined as the binding index (BI), and it is calculated as shown in Equation (1):

BI = (NI1 + NI2 + NI3)/(AI1 + AI2 + AI3)(1)

where AI1, AI2, and AI3 represent the areas of three different regions, while NI1, NI2, and NI3 represent the number of sperm bound to each of these regions.

### 2.12. Statistical Analysis

All statistical analyses were performed using IBM SPSS Statistics version 27 (IBM Corp., Armonk, NY, USA) and R software (version 4.3.3; R Foundation for Statistical Computing, Vienna, Austria). Data were first tested for normality and homogeneity of variance. Differences among groups were analyzed using one-way ANOVA, and independent-sample *t*-tests were applied for pairwise comparisons. Statistical analyses for Tables S1 and S2 were performed in R (version 4.3.3) using the tidyverse (version 2.0.0), rstatix (version 0.7.2), multcompView (version 0.1.10), flextable (version 0.9.9), and officer (version 0.6.10) packages. One-way ANOVA followed by Tukey’s post hoc test was used to assess differences among NMN concentration groups, with significant differences indicated by distinct superscript letters (*p* < 0.05). ImageJ software (version 1.54; National Institutes of Health, USA) was used for the grayscale analysis of Western blot bands. Flow cytometry data (.FCS files) were analyzed using FlowJo software (version 10.8.1; BD Biosciences, USA), with identical gating strategies applied across all samples. Data visualization and graph preparation were performed using GraphPad Prism version 8.0 (GraphPad Software Inc., La Jolla, CA, USA). Results are expressed as the mean ± standard error of the mean (SEM), and a *p* < 0.05 was considered statistically significant.

## 3. Results

### 3.1. NMN Enhanced Sperm Motility and Viability and Acrosome Integrity During Liquid Storage

**Figure 1:**
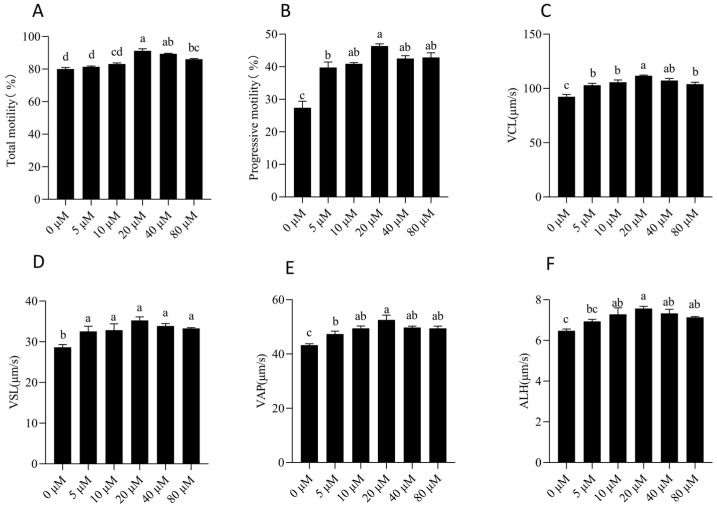
Effects of different NMN concentrations on boar sperm motility parameters after 7 days of storage. (**A**–**F**) Dynamic changes in sperm parameters. Statistical comparisons among groups were conducted using one-way ANOVA followed by Tukey’s post hoc test. Values are expressed as mean ± SEM (*n* = 3). Different lowercase letters (a–c) represent significant differences (*p* < 0.05). VCL: curvilinear velocity; VSL: straight-line velocity; VAP: average path velocity; ALH: amplitude of lateral head displacement.

As shown in Figure 1 and Table S1, NMN supplementation influenced the temporal dynamics of boar sperm motility during 7 days of storage. Sperm total motility at time points of 5 and 7 days showed a significant improvement in the NMN-treated group when compared to the control (*p* < 0.05). Regarding kinematic parameters, the sperm progressive motility, curvilinear velocity (VCL), straight-line velocity (VSL), average path velocity (VAP), and amplitude of lateral head displacement (ALH) in the 20 μM NMN group were also significantly higher than those in the control during sperm storage from 3 days to 7 days (*p* < 0.05). In contrast, other parameters, including straightness (STR), linearity (LIN), wobble (WOB), and beat-cross frequency (BCF), did not show significant differences between groups at any time points (*p* > 0.05).

**Figure 2:**
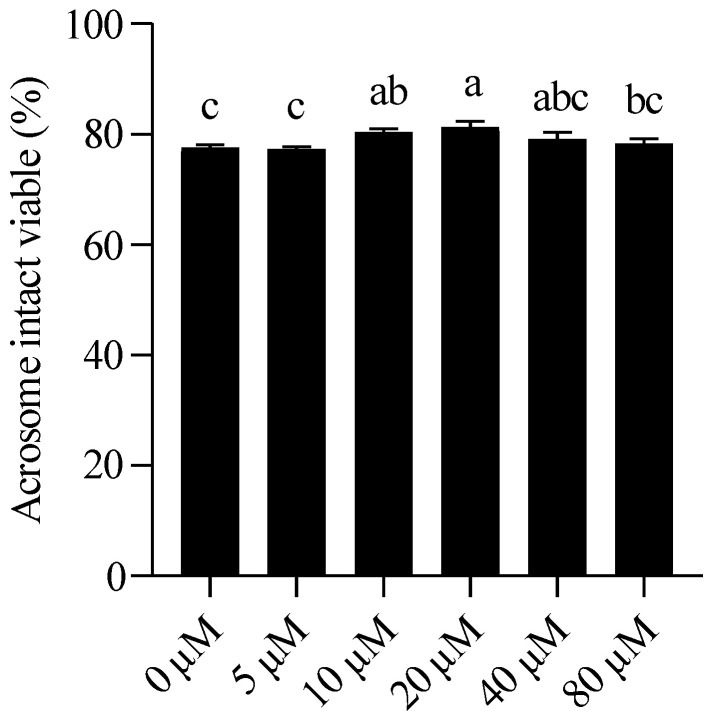
Boar sperm viability and acrosome integrity are maintained by NMN supplementation after 7 days of storage. Statistical comparisons among groups were conducted using one-way ANOVA followed by Tukey’s post hoc test. Values are expressed as mean ± SEM (*n* = 3). Different lowercase letters (a–c) represent significant differences (*p* < 0.05).

As shown in Figure 2 and Supplementary Figures S1 and S2, the percentage of acrosome-intact viable sperm was significantly higher in the NMN-treated groups than in the control group from 3 days of storage onward, with the difference being most pronounced on 7 days. The protective effect of low concentrations of NMN (5–10 μM) declined over time, whereas supplementation with 20 μM NMN consistently maintained higher acrosomal integrity throughout the storage period.

### 3.2. NMN Improved Sperm Thermo-Resistance After Storage

**Figure 3:**
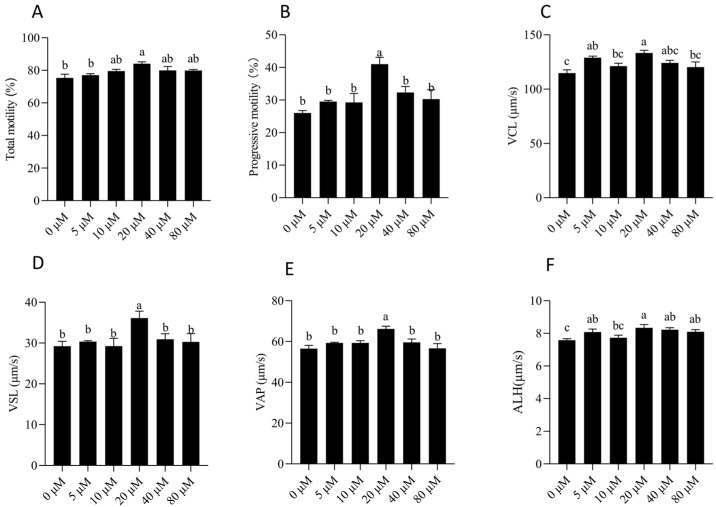
Thermotolerance of boar sperm after 7 days of storage in extenders supplemented with different NMN concentrations. (**A**–**F**) Dynamic changes of 7 days storage sperm parameters after 5 h of incubation at 37 °C. Statistical comparisons among groups were conducted using one-way ANOVA followed by Tukey’s post hoc test. Values are expressed as mean ± SEM (*n* = 3). Different lowercase letters (a–c) represent significant differences (*p* < 0.05). VCL: curvilinear velocity; VSL: straight-line velocity; VAP: average path velocity; ALH: amplitude of lateral head displacement.

As shown in Figure 3 and Table S2, NMN supplementation influenced sperm motility performance during the thermo-resistance (TRT) test. After 5 h of incubation, total motility (TM), progressive motility (PM), curvilinear velocity (VCL), straight-line velocity (VSL), average path velocity (VAP), wobble (WOB), and amplitude of lateral head displacement (ALH) were all significantly higher in the 20 μM NMN group compared with the control (*p* < 0.05). In contrast, no significant differences were observed in the remaining kinematic parameters between groups (*p* > 0.05). These results indicate that supplementation with 20 μM NMN effectively preserves sperm kinetic performance under thermal challenge.

### 3.3. NMN Preserved Boar Sperm Mitochondrial Membrane Potential

**Figure 4:**
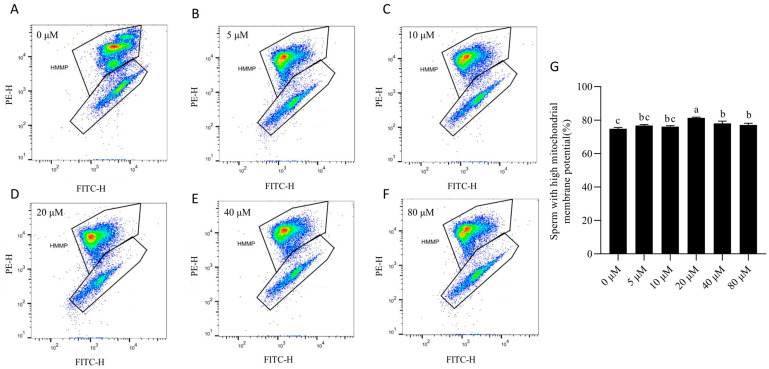
Proportion of boar sperm with high mitochondrial membrane potential (MMP) after 7 days of storage in extenders supplemented with NMN. (**A**–**F**) Flow cytometry evaluation of sperm mitochondrial membrane potential (MMP); (**G**) sperm MMP. Sperm with high MMP (HMMP) are represented in the upper gate, while the lower gate represents sperm with low MMP. Statistical analysis was performed using one-way ANOVA followed by Tukey’s post hoc test. Values are presented as mean ± SEM (*n* = 3). Different lowercase letters (a–c) represent significant differences (*p* < 0.05).

Mitochondrial membrane potential (MMP) was markedly reduced in the control group after 7 days of storage (Figure 4). Supplementation with 20 μM NMN significantly (*p* < 0.05) restored MMP level, with a comparable effect observed at 40 μM. In contrast, lower concentrations (5–10 μM) showed no significant improvement compared with the control, and supplementing the extender with 80 μM NMN did not provide additional benefits.

### 3.4. NMN Supplementation Improved Mitochondrial Metabolic Activity of Boar Sperm by Maintaining NAD^+^ Homeostasis

**Figure 5:**
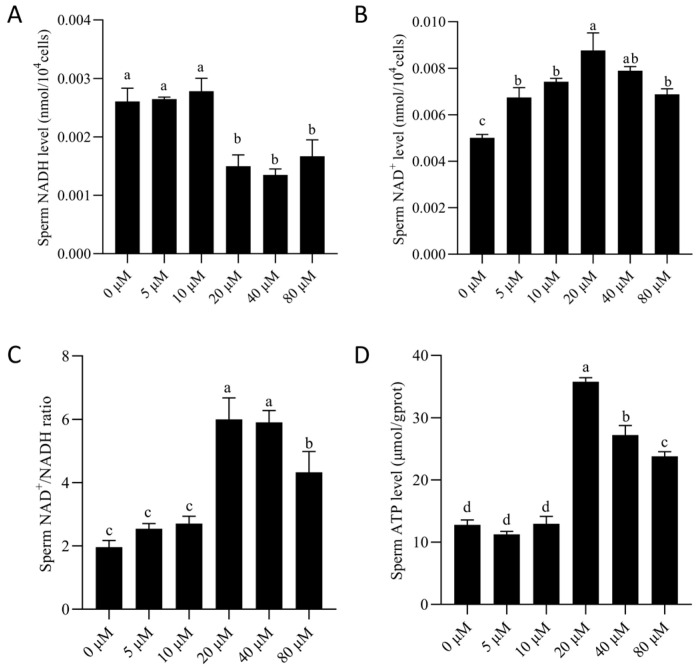
Effect of NMN supplementation on intracellular energy metabolism in boar sperm during storage. (**A**) Intracellular ATP level; (**B**) Intracellular NAD^+^ level; (**C**) Intracellular NADH level; (**D**) NAD^+^/NADH ratio. Values are presented as mean ± SEM (*n* = 3). Statistical analysis was performed using one-way ANOVA followed by Tukey’s post hoc test. Different lowercase letters (a–c) represent significant differences (*p* < 0.05).

As shown in Figure 5, NMN supplementation significantly (*p* < 0.05) elevated ATP level in boar sperm after 7 days of storage, with significant increases observed at 20 and 40 μM. Intracellular NAD^+^ level also increased, while NADH level declined markedly from 20 μM onwards. Accordingly, the NAD^+^/NADH ratio exhibited an upward trend similar to ATP level.

### 3.5. Expression of NMNAT3 in Boar Sperm

**Figure 6:**
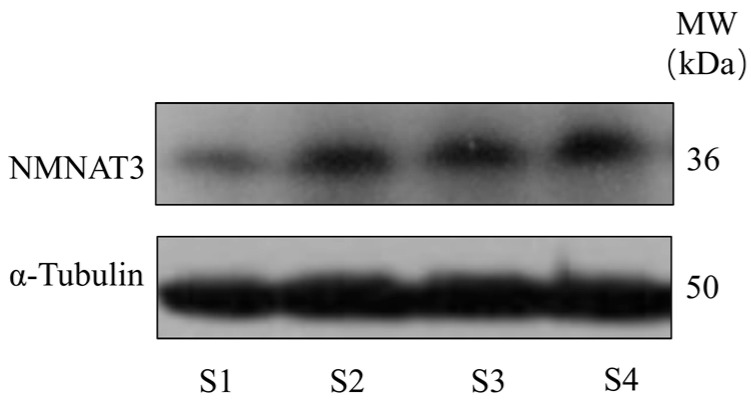
Expression of NMNAT3 in boar sperm. S1: sperm from boar 1; S2: sperm from boar 2; S3: sperm from boar 3; S4: sperm from boar 4.

Western blot analysis detected a clear 36-kDa band of NMNAT3 in boar sperm (Figure 6), confirming its presence in mature sperm. The detection of NMNAT3 suggests that boar sperm retain the enzymatic capacity for NAD^+^ synthesis, supporting the observed improvement in NAD^+^ levels and sperm function following NMN supplementation.

### 3.6. NMN Maintained Boar Sperm Fertility Ability During Liquid Storage

**Figure 7:**
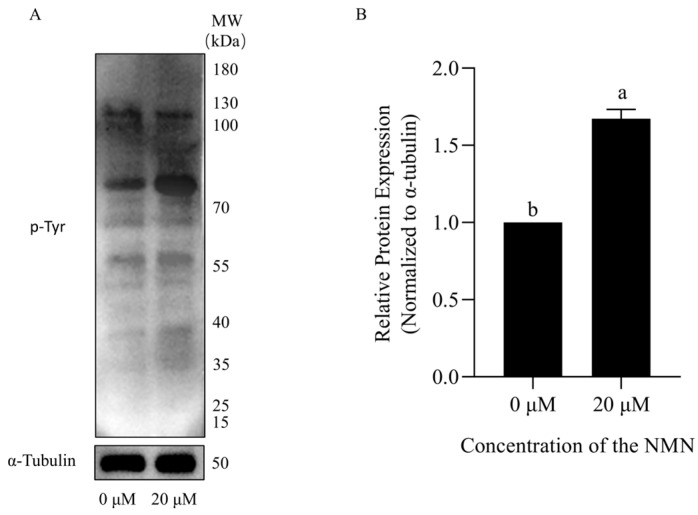
Tyrosine phosphorylation in capacitated sperm after preservation with NMN for 7 days. (**A**) Representative immunoblot of tyrosine-phosphorylated proteins in sperm. A molecular weight marker (kDa) is indicated on the right. (**B**) Band intensities were normalized to the α-Tubulin loading control and were expressed relative to the control group (0 μM NMN), which was set as 1.0. Statistical analysis was determined by an independent samples *t*-test. Values are presented as mean ± SEM (*n* = 3). Different lowercase letters (a, b) represent significant differences (*p* < 0.05).

**Figure 8:**
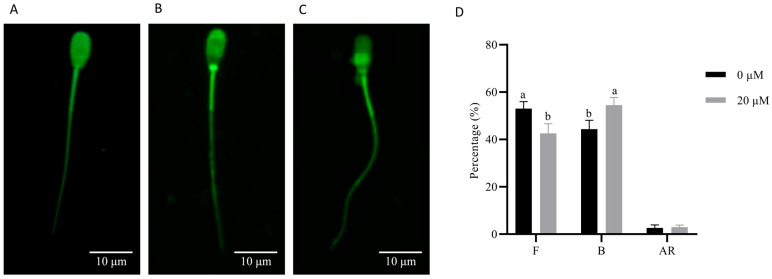
CTC staining analysis of capacitation status in boar sperm after 7 days of storage. (**A**) F, uncapacitated; (**B**) B, capacitated; (**C**) AR, acrosome-reacted. (**D**) Percentage of sperm with and without capacitation. Statistical analysis was determined by an independent samples *t*-test. Values are presented as mean ± SEM (*n* = 3). Different lowercase letters (a, b) represent significant differences (*p* < 0.05).

To further evaluate the effect of NMN on boar sperm function after preservation, the sperm capacitation was evaluated. It was observed that the tyrosine phosphorylation level in 20 μM NMN-treated sperm was significantly (*p* < 0.05) higher than that in the control group after induced capacitation (Figure 7). In addition, there are three distinct capacitation states of boar sperm after CTC staining (Figure 8A–C). The F pattern (uncapacitated) was characterized by uniform fluorescence across the entire sperm head, indicating intact plasma membrane and absence of capacitation. The B pattern (capacitated) showed a fluorescence-free band in the post-acrosomal region, reflecting capacitation-associated membrane changes. The AR pattern (acrosome-reacted) was defined by the absence of fluorescence over the acrosomal region, indicating acrosome exocytosis. Furthermore, CTC staining demonstrated that the capacitation rate of boar sperm after 7 days of liquid storage was significantly higher in the 20 μM NMN group than in the control group (*p* < 0.05; Figure 8D).

### 3.7. NMN Improved the Binding Capacity of Sperm Stored for 7 Days to Oviduct Explants

**Figure 9:**
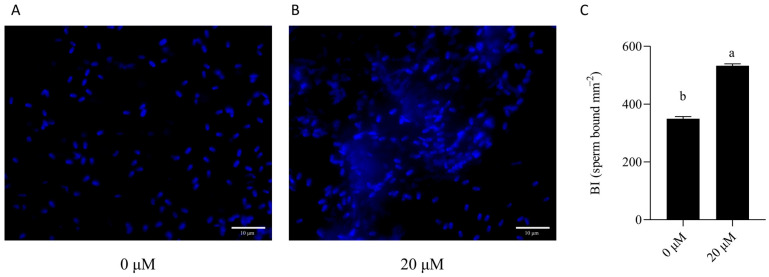
Effect of NMN supplementation on the post-storage binding capacity of boar sperm to oviductal explants. (**A**) Control group; (**B**) 20 μM NMN group. Representative images showing sperm bound to the oviductal epithelial surface after 7 days of liquid storage (scale bar = 10 μm); (**C**) sperm binding index (BI), Statistical analysis was determined by an independent samples *t*-test. Values are presented as mean ± SEM (*n* = 3). Different lowercase letters (a, b) represent significant differences (*p* < 0.05).

To evaluate the effect of NMN supplementation in the semen extender on the fertilization capacity of boar sperm after 7 days of liquid storage, Hoechst 33342 staining was performed, and the binding index (BI) was used to assess sperm reservoir formation ability in the oviduct. As shown in Figure 9, sperm BI in the 20 μM NMN group was significantly higher than that in the control group (*p* < 0.05), indicating that NMN supplementation enhances the reservoir-forming potential of stored sperm.

## 4. Discussion

In the present study, NMN supplementation markedly preserved sperm motility parameters during liquid storage, including total motility, progressive motility, and straight-line velocity. These results are in agreement with previous studies in mice and sheep, where NMN was reported to improve sperm quality by maintaining mitochondrial integrity and energy production [29,30]. The consistency across species suggests that NMN acts as a conserved metabolic regulator of sperm function. Given the crucial role of motility in successful fertilization, these findings highlight the physiological relevance of NMN in sustaining sperm viability during storage. Overall, our findings showed that supplementing the Modena extender at a concentration of 20 μM NMN enhanced sperm motility parameters and improved mitochondrial function. These results are in contrast with the findings of Zhang et al. [21], who reported an optimal effect of NMN at a concentration of 150 μM in boar semen preservation when it was diluted with BTS extender. The difference might be due to the different boar semen extenders that are composed of different substrates.

Thermo-resistance testing was applied to simulate the physiological environment of the female reproductive tract, where sperm must remain viable for several hours before fertilization. Consistent with previous studies [31], thermo-resistance reflects the metabolic competence and mitochondrial performance of sperm. Our results show that NMN supplementation markedly enhances thermo-resistance after 7 days of storage, indicating improved cellular stability under elevated temperature. This improvement may result from NMN-mediated support of mitochondrial function and intracellular NAD^+^ availability. Importantly, the enhanced thermo-resistance was consistent with our oviduct explant assay results, in which NMN-treated sperm exhibited better binding capacity. Taken together, these results suggest that NMN may help maintain the fertilization potential of boar sperm during extended storage.

Mitochondria are the core determinants of sperm energy metabolism and motility [32], and NAD^+^ plays an essential role in sustaining mitochondrial ATP production. In this study, NMN supplementation increased the NAD^+^/NADH ratio and ATP level, suggesting improved mitochondrial metabolic efficiency during liquid storage [33]. The detection of NMNAT3 further indicates that NMN may support NAD^+^ turnover through this mitochondrial pathway [34,35], potentially counteracting storage-related NAD^+^ decline, which has been associated with CD38 activity and mitochondrial impairment [36]. Although mitochondrial structure was not directly assessed, the observed enhancement in sperm motility and viability is consistent with improved mitochondrial function, aligning with previous findings that mitochondrial integrity and ATP generation are key determinants of fertilizing potential [37,38]. As a direct NAD^+^ precursor, NMN elevates intracellular NAD^+^, promotes electron flow through respiratory chain complex I, and improves ATP generation efficiency [34,39], which may collectively support flagellar motion, capacitation, and acrosome reaction. Overall, these findings suggest that NMN maintains sperm function primarily by stabilizing NAD^+^ metabolism and supporting mitochondrial energy production during extended liquid storage.

Sperm capacitation involves a series of physiological and biochemical transformations required for the acquisition of fertilization competence. This process is energetically demanding, relying on ATP supplied through both glycolysis and mitochondrial activity. Previous metabolomic studies have demonstrated a shift toward elevated lactate, phosphate, and polyol intermediates during capacitation, accompanied by a decline in glucose and citrate, reflecting redistribution of metabolic flux to support membrane remodeling and motility-associated processes [40]. In this context, NMN supplementation appears to strengthen the energy supply by supporting mitochondrial activity and overall metabolic function. Consistent with this, sperm from the NMN-treated group exhibited higher levels of protein tyrosine phosphorylation—a biochemical hallmark of capacitation [41], as well as an increased proportion of capacitated sperm based on CTC staining. These findings indicate that NMN enhances both the molecular signaling events and functional maturation associated with capacitation, potentially through improved ATP availability and metabolic efficiency. Notably, NMN preserved capacitation potential even after 7 days of liquid storage, suggesting that it helps maintain membrane integrity and metabolic stability during prolonged preservation. Since capacitation is closely associated with fertilizing ability and is a critical predictor of boar reproductive performance, despite normal motility, up to 25% of boars still exhibit conception rates below 80% [42]. Our results support the potential value of NMN as an effective additive for maintaining fertilization potential in artificial insemination programs.

## 5. Conclusions

This study demonstrates that exogenous NMN supplementation effectively maintains the quality and function of boar sperm during liquid storage. Notably, NMN at a concentration of 20 μM improved the quality of boar sperm stored in vitro, indicating a protective effect at a relatively low dose. NMN treatment significantly enhanced post-storage sperm parameters, maintaining total and progressive motility and better preserving acrosome integrity. In addition, NMN-treated sperm showed higher mitochondrial membrane potential and increased ATP and NAD^+^ level, reflecting sustained energy metabolism. These cellular improvements translated into greater fertilization potential, as evidenced by elevated protein tyrosine phosphorylation, higher capacitation rates defined by CTC patterns, and improved sperm storage sac formation. Collectively, these findings show that NMN preserves sperm function during in vitro liquid storage by supporting mitochondrial activity and energy metabolism, providing new insights for optimizing semen preservation strategies in swine reproduction.

## Data Availability

The data supporting the findings of this study are included in this article and its Supplementary Materials. Additional data are available from the corresponding author upon reasonable request.

## Supplementary Materials

The following supporting information can be downloaded at: https://www.mdpi.com/article/10.3390/ani15233383/s1, Table S1: Effects of different NMN concentrations on boar sperm motility during storage. Table S2: Thermotolerance of boar sperm after 7 days of storage in extenders supplemented with different NMN concentrations. Figures S1 and S2: Flow cytometry analysis of the effect of NMN supplementation in extender on boar sperm viability and acrosome integrity.
