# Supplementary material for: Nicotinamide Mononucleotide Enhances Boar Sperm Quality via Maintaining Mitochondrial Function During Liquid Storage
Source: Animals (Basel). 2025 Nov 22;15(23):3383. doi: 10.3390/ani15233383 (PMC12691309; doi:10.3390/ani15233383)
Supplement: Supplementary file 1 [file animals-15-03383-s001.zip › animals-3968884-supplementary.pdf]

Supplementary Material

1. Supplementary Tables

Table S1. Effects of different NMN concentrations on boar sperm motility during storage.

Parameters	Days	0 μM	5 μM	10 μM	20 μM	40 μM	80 μM
Total motility(%)	1	97.6 ± 0.5	97.4 ± 0.6	97.9 ± 0.2	97.8 ± 0.3	97.2 ± 1.0	98.2 ± 0.2
	3	91.0 ± 0.5	92.8 ± 1.3	93.1 ± 1.0	94.3 ± 0.7	94.7 ± 0.2	93.6 ± 1.2
	5	86.2 ± 0.7^{b}	89.9 ± 1.6^{a}	90.3 ± 1.0^{a}	92.6 ± 1.2^{a}	90.5 ± 0.8^{a}	89.3 ± 0.9^{ab}
	7	80.1 ± 0.9^{d}	81.4 ± 0.5^{cd}	83.1 ± 0.7^{c}	91.2 ± 1.2^{a}	89.4 ± 0.3^{a}	86.0 ± 0.4^{b}
Progressive motility(%)	1	52.1 ± 0.7	48.9 ± 0.9	52.8 ± 0.7	51.3 ± 0.6	50.4 ± 1.2	49.7 ± 1.4
	3	37.8 ± 0.5^{b}	51.0 ± 2.8^{a}	54.0 ± 1.1^{a}	53.1 ± 1.5^{a}	53.9 ± 1.5^{a}	52.0 ± 1.7^{a}
	5	36.1 ± 3.0^{c}	42.9 ± 1.2^{b}	40.9 ± 2.6^{bc}	50.8 ± 1.1^{a}	43.8 ± 0.5^{b}	41.6 ± 0.5^{bc}
	7	27.4 ± 2.0^{c}	39.8 ± 1.7^{b}	40.9 ± 0.4^{b}	46.4 ± 0.6^{a}	42.5 ± 0.9^{ab}	42.9 ± 1.4^{ab}
VCL(μm/s)	1	110.7 ± 2.6	107.5 ± 7.1	123.5 ± 2.6	120.0 ± 1.7	115.5 ± 1.9	118.7 ± 2.4
	3	95.6 ± 2.2^{b}	112.1 ± 4.3^{a}	115.5 ± 2.2^{a}	122.1 ± 3.9^{a}	112.5 ± 3.7^{a}	120.9 ± 3.1^{a}
	5	96.7 ± 1.4^{c}	104.9 ± 0.5^{b}	106.3 ± 1.4^{b}	112.3 ± 0.9^{a}	110.4 ± 0.0^{a}	106.2 ± 1.9^{b}
	7	92.3 ± 2.1^{c}	102.9 ± 1.8^{b}	105.6 ± 2.1^{b}	111.7 ± 0.6^{a}	107.3 ± 1.9^{ab}	103.9 ± 1.7^{b}
VSL(μm/s)	1	35.3 ± 0.7	35.2 ± 1.8	37.2 ± 0.8	36.5 ± 0.3	35.1 ± 0.4	36.2 ± 0.4
	3	30.7 ± 0.5^{b}	33.8 ± 0.9^{a}	34.1 ± 0.3^{a}	36.3 ± 0.7^{a}	35.3 ± 1.1^{a}	35.5 ± 0.6^{a}
	5	29.7 ± 0.5^{c}	32.3 ± 0.1^{ab}	32.1 ± 1.2^{b}	34.3 ± 0.4^{a}	32.9 ± 0.3^{ab}	32.5 ± 0.5^{abc}
	7	28.6 ± 0.7^{b}	32.5 ± 1.3^{a}	32.9 ± 1.6^{a}	35.2 ± 0.9^{a}	33.9 ± 0.6^{a}	33.3 ± 0.2^{a}
VAP(μm/s)	1	55.2 ± 1.1	54.5 ± 3.7	61.7 ± 1.0	61.0 ± 0.9	57.9 ± 1.1	59.7 ± 1.0
	3	45.9 ± 1.0^{b}	54.4 ± 1.9^{a}	55.0 ± 0.4^{a}	58.1 ± 1.3^{a}	54.4 ± 1.6^{a}	57.3 ± 0.7^{a}
	5	45.1 ± 1.4^{c}	49.6 ± 0.5^{b}	49.4 ± 1.2^{b}	53.5 ± 0.7^{a}	53.5 ± 0.2^{a}	48.8 ± 1.0^{b}
	7	43.3 ± 0.5^{c}	47.4 ± 1.0^{b}	49.5 ± 0.9^{ab}	52.5 ± 1.8^{a}	49.8 ± 0.5^{ab}	49.5 ± 0.8^{ab}
STR(%)	1	63.9 ± 2.1	63.7 ± 1.5	59.7 ± 0.7	59.5 ± 0.4	58.7 ± 0.8	59.7 ± 1.8
	3	65.8 ± 0.5	61.4 ± 0.8	61.6 ± 1.0	62.2 ± 0.8	65.0 ± 3.4	61.2 ± 0.4
	5	65.0 ± 0.6	65.6 ± 1.0	64.8 ± 3.3	63.5 ± 0.7	61.7 ± 0.5	65.7 ± 1.5
	7	66.0 ± 1.7	66.5 ± 1.4	65.1 ± 2.1	64.5 ± 0.3	67.4 ± 1.8	66.9 ± 1.5
LIN(%)	1	32.7 ± 1.1	33.1 ± 0.7	30.5 ± 0.6	30.9 ± 0.2	30.3 ± 0.5	30.5 ± 1.0
	3	32.5 ± 0.5	31.2 ± 0.8	30.4 ± 0.7	30.6 ± 0.5	32.3 ± 1.6	29.7 ± 0.3
	5	31.0 ± 0.9	32.3 ± 0.9	30.5 ± 1.0	30.9 ± 0.4	30.4 ± 0.2	31.9 ± 0.9
	7	32.1 ± 1.3	32.0 ± 0.5	31.6 ± 1.3	31.8 ± 0.7	32.9 ± 1.1	32.6 ± 0.5
WOB(%)	1	50.4 ± 0.3	50.9 ± 0.1	50.1 ± 0.3	51.0 ± 0.3	50.4 ± 0.2	50.4 ± 0.4
	3	48.7 ± 0.4	49.7 ± 0.6	48.7 ± 0.8	48.6 ± 0.4	48.9 ± 0.2	47.7 ± 0.7
	5	47.2 ± 1.2	48.4 ± 0.6	46.9 ± 0.8	48.4 ± 0.2	48.9 ± 0.1	47.6 ± 0.6
	7	47.9 ± 0.7	47.0 ± 0.5	47.7 ± 0.6	48.0 ± 1.1	47.9 ± 0.4	48.4 ± 0.2
ALH (μm)	1	7.4 ± 0.2	7.6 ± 0.3	8.2 ± 0.1	8.1 ± 0.2	7.5 ± 0.2	8.0 ± 0.1
	3	6.9 ± 0.2^{b}	7.6 ± 0.2^{b}	7.9 ± 0.2^{ab}	8.4 ± 0.1^{a}	7.8 ± 0.3^{ab}	8.5 ± 0.2^{a}
	5	7.1 ± 0.2^{c}	7.4 ± 0.3^{ab}	7.4 ± 0.1^{ab}	7.8 ± 0.2^{a}	7.9 ± 0.1^{a}	7.0 ± 0.2^{c}
	7	6.5 ± 0.1^{c}	6.9 ± 0.1^{bc}	7.3 ± 0.3^{ab}	7.6 ± 0.1^{a}	7.3 ± 0.2^{ab}	7.1 ± 0.1^{ab}
BCF (Hz)	1	32.2 ± 0.3	32.7 ± 0.6	32.4 ± 0.6	32.1 ± 1.4	32.5 ± 1.2	31.8 ± 0.9
	3	28.4 ± 0.7	30.1 ± 0.5	29.2 ± 0.3	28.6 ± 0.3	29.6 ± 0.5	29.6 ± 1.0
	5	28.1 ± 0.8	29.0 ± 1.6	27.0 ± 1.1	27.7 ± 0.8	27.3 ± 0.4	30.6 ± 3.2
	7	26.4 ± 1.8	27.4 ± 1.3	29.5 ± 0.6	30.2 ± 3.4	24.6 ± 0.7	27.0 ± 1.1

Statistical comparisons among groups were conducted using one-way ANOVA followed by Tukey's post hoc test (n = 3). Results are expressed as mean ± SEM from five independent experiments. Different superscript letters (a–d) indicate significant differences among mean values within the same parameter ($p < 0.05$). Abbreviations for sperm kinematic parameters are as follows: VCL, curvilinear velocity; VSL,

straight-line velocity; VAP, average path velocity; BCF, beat-cross frequency; ALH, amplitude of lateral head displacement; STR (VSL/VAP), straightness; LIN (VSL/VCL), linearity; WOB (VAP/VCL), wobble. a,b,c,d Different letters indicate significant differences between mean values for a given behavior ($p < 0.05$).

Table S2. Thermotolerance of boar sperm after 7 days storage in extenders supplemented with different NMN concentrations.

Parameters	Time (h)	0 μM	5 μM	10 μM	20 μM	40 μM	80 μM
	1	86.1 ± 1.4 ^{b}	86.0 ± 0.9^{b}	90.4 ± 1.3^{ab}	93.1 ± 0.8^{a}	90.4 ± 1.3^{a}	91.6 ± 1.2^{a}
	2	86.2 ± 2.6^{b}	87.7 ± 0.7^{b}	90.5 ± 1.1^{ab}	94.2 ± 0.7^{a}	89.4 ± 1.7^{b}	87.6 ± 0.7^{b}
Total motility (%)	3	86.3 ± 1.8	85.1 ± 1.1	88.6 ± 0.9	90.5 ± 1.9	87.7 ± 0.6	85.6 ± 0.7
	4	82.3 ± 0.9^{b}	81.9 ± 0.7^{b}	86.6 ± 1.1^{a}	89.9 ± 1.9^{a}	87.5 ± 1.2^{a}	86.4 ± 1.0^{a}
	5	75.4 ± 2.3^{b}	77.0 ± 0.9^{b}	79.5 ± 1.1^{ab}	84.0 ± 1.2^{a}	80.0 ± 2.4^{ab}	79.9 ± 0.7^{ab}
	1	30.1 ± 2.7^{b}	30.6 ± 0.4^{b}	35.3 ± 1.5^{b}	40.8 ± 1.1^{a}	31.7 ± 1.6^{b}	30.7 ± 1.1^{b}
	2	31.5 ± 0.3	31.6 ± 2.5	35.4 ± 2.1	39.3 ± 3.7	35.7 ± 1.3	37.6 ± 2.0
Progressive motility (%)	3	36.5 ± 1.6^{bc}	33.4 ± 1.9^{c}	39.5 ± 1.5^{b}	45.5 ± 2.2^{a}	35.5 ± 0.5^{bc}	31.1 ± 2.2^{c}
	4	35.3 ± 3.9^{bc}	31.1 ± 2.5^{c}	41.2 ± 0.6^{ab}	45.7 ± 1.6^{a}	37.4 ± 0.1^{bc}	36.6 ± 2.6^{bc}
	5	26.0 ± 0.8^{b}	29.5 ± 0.4^{b}	29.3 ± 2.7^{b}	41.0 ± 2.1^{a}	32.3 ± 1.9^{b}	30.3 ± 2.9^{b}
	1	112.5 ± 3.3^{c}	126.4 ± 0.3^{b}	125.3 ± 2.6^{b}	140.6 ± 3.0^{a}	128.2 ± 3.9^{b}	120.4 ± 4.8^{bc}
	2	125.1 ± 4.9	130.4 ± 4.2	136.7 ± 1.6	134.3 ± 7.9	138.2 ± 5.5	116.3 ± 3.3
VCL (μm/s)	3	133.3 ± 4.6	132.5 ± 3.6	133.9 ± 5.5	144.3 ± 1.8	126.2 ± 3.0	124.2 ± 6.8
	4	118.6 ± 4.5	119.9 ± 4.2	137.3 ± 4.9	125.9 ± 1.7	119.6 ± 3.0	135.8 ± 9.8
	5	114.8 ± 3.0^{c}	129.0 ± 1.5^{ab}	121.2 ± 2.7^{bc}	133.3 ± 2.5^{a}	124.2 ± 2.4^{abc}	120.3 ± 4.8^{bc}
	1	28.1 ± 1.5^{c}	31.2 ± 0.3^{bc}	32.7 ± 1.4^{b}	36.2 ± 0.2^{a}	31.2 ± 0.3^{bc}	29.7 ± 1.4^{bc}
	2	31.5 ± 1.4	30.8 ± 0.9	34.1 ± 1.2	34.0 ± 2.4	34.5 ± 1.7	31.9 ± 0.4
VSL (μm/s)	3	36.4 ± 0.1^{ab}	33.0 ± 0.7^{cd}	35.8 ± 1.3^{abc}	39.0 ± 0.4^{a}	33.2 ± 1.3^{bcd}	30.8 ± 1.4^{d}
	4	33.4 ± 1.9^{ab}	29.3 ± 0.9^{b}	37.2 ± 1.3^{a}	35.5 ± 0.9^{a}	33.8 ± 1.1^{ab}	34.0 ± 2.0^{ab}
	5	29.2 ± 1.2^{b}	30.4 ± 0.2^{b}	29.2 ± 1.9^{b}	36.1 ± 1.7^{a}	30.9 ± 1.4^{b}	30.3 ± 2.0^{b}
	1	54.4 ± 1.2^{c}	61.6 ± 0.4^{b}	62.4 ± 1.6^{ab}	67.5 ± 1.6^{a}	63.4 ± 1.7^{ab}	59.2 ± 3.0^{bc}
	2	61.0 ± 2.5	63.4 ± 1.9	65.4 ± 1.6	65.7 ± 4.5	65.9 ± 2.8	57.9 ± 1.9
VAP (μm/s)	3	63.9 ± 1.6^{bc}	63.4 ± 1.7^{bc}	64.8 ± 2.5^{ab}	70.9 ± 0.3^{a}	59.9 ± 2.0^{bc}	57.3 ± 3.3^{c}
	4	59.8 ± 2.4	58.3 ± 1.7	68.3 ± 2.8	62.9 ± 1.2	60.6 ± 2.1	64.4 ± 4.2
	5	56.5 ± 1.6^{b}	59.3 ± 0.3^{b}	59.3 ± 1.1^{b}	66.2 ± 1.3^{a}	59.6 ± 1.7^{b}	56.7 ± 2.3^{b}
	1	51.7 ± 1.6^{ab}	51.2 ± 0.6^{ab}	53.4 ± 1.4^{a}	53.1 ± 0.6^{a}	48.7 ± 1.4^{b}	49.1 ± 0.4^{b}
	2	51.3 ± 0.2^{b}	48.8 ± 0.7^{b}	51.4 ± 1.1^{b}	51.7 ± 1.0^{b}	50.8 ± 0.5^{b}	55.2 ± 2.0^{a}
STR (%)	3	54.3 ± 0.6	51.4 ± 0.5	53.2 ± 0.5	53.6 ± 0.5	52.6 ± 0.7	52.7 ± 0.5
	4	55.0 ± 1.8^{a}	49.6 ± 1.2^{b}	53.5 ± 0.5^{a}	55.0 ± 1.8^{a}	54.8 ± 0.3^{a}	52.1 ± 0.6^{ab}
	5	51.3 ± 0.7	50.1 ± 0.5	50.6 ± 1.8	52.7 ± 0.9	50.7 ± 0.7	52.4 ± 0.3
	1	25.7 ± 0.4^{b}	25.7 ± 0.3^{b}	27.3 ± 0.7^{a}	26.2 ± 0.2^{ab}	25.1 ± 0.8^{b}	24.6 ± 0.1^{b}
	2	25.5 ± 0.2^{b}	24.4 ± 0.6^{b}	25.5 ± 0.3^{b}	25.9 ± 0.4^{b}	24.7 ± 0.4^{b}	28.4 ± 1.1^{a}
LIN (%)	3	26.6 ± 0.7	25.1 ± 0.2	26.3 ± 0.3	26.8 ± 0.6	25.8 ± 0.8	25.2 ± 0.3
	4	28.7 ± 1.2^{a}	24.7 ± 1.0^{b}	27.1 ± 0.3^{ab}	27.9 ± 0.8^{a}	28.4 ± 0.5^{a}	25.2 ± 0.6^{b}
	5	25.7 ± 0.3	24.0 ± 0.5	25.7 ± 1.1	27.0 ± 0.6	25.2 ± 0.4	25.2 ± 1.0
	1	48.5 ± 0.6	48.8 ± 0.4	50.1 ± 0.4	47.9 ± 0.2	49.6 ± 0.6	48.9 ± 0.4
	2	48.5 ± 0.2^{b}	48.8 ± 0.4^{ab}	48.2 ± 0.6^{b}	48.8 ± 0.4^{ab}	47.7 ± 0.4^{b}	50.0 ± 0.4^{a}
WOB (%)	3	47.6 ± 1.0	47.6 ± 0.1	48.4 ± 0.5	49.1 ± 0.4	47.9 ± 0.8	46.5 ± 0.2
	4	50.5 ± 0.3^{a}	48.4 ± 0.5^{bc}	49.5 ± 0.3^{ab}	49.5 ± 0.2^{ab}	50.5 ± 0.6^{a}	47.2 ± 0.6^{c}
	5	48.9 ± 0.2^{a}	46.0 ± 0.7^{c}	49.6 ± 0.5^{a}	49.4 ± 0.1^{a}	48.2 ± 0.3^{ab}	46.9 ± 1.2^{bc}
	1	7.5 ± 0.0^{b}	8.0 ± 0.1^{ab}	7.9 ± 0.1^{ab}	8.8 ± 0.3^{a}	8.2 ± 0.3^{b}	8.0 ± 0.1^{ab}
	2	8.2 ± 0.3^{bc}	8.4 ± 0.3^{b}	8.3 ± 0.1^{b}	8.6 ± 0.3^{ab}	9.4 ± 0.3^{a}	7.5 ± 0.1^{c}
ALH (μm)	3	8.7 ± 0.3	8.3 ± 0.2	8.6 ± 0.1	8.5 ± 0.1	8.9 ± 0.2	8.2 ± 0.4
	4	7.7 ± 0.2^{b}	7.6 ± 0.3^{b}	8.6 ± 0.2^{a}	8.0 ± 0.1^{ab}	7.7 ± 0.1^{b}	8.5 ± 0.3^{a}
	5	7.6 ± 0.1^{c}	8.1 ± 0.2^{ab}	7.7 ± 0.2^{bc}	8.3 ± 0.2^{a}	8.2 ± 0.1^{ab}	8.1 ± 0.1^{ab}

Parameters	Time (h)	0 μM	5 μM	10 μM	20 μM	40 μM	80 μM
	1	34.9 ± 0.1	34.8 ± 2.1	33.4 ± 1.1	34.8 ± 0.5	37.2 ± 0.3	35.2 ± 0.6
	2	34.8 ± 0.1	35.1 ± 1.0	36.1 ± 0.3	34.9 ± 0.3	34.6 ± 0.3	33.5 ± 0.5
BCF (Hz)	3	35.5 ± 0.7	36.1 ± 1.1	36.4 ± 0.6	35.7 ± 0.4	35.1 ± 1.1	34.9 ± 0.2
	4	35.9 ± 0.7	36.3 ± 0.2	35.1 ± 0.9	36.2 ± 0.3	35.5 ± 0.8	36.6 ± 0.7
	5	35.6 ± 0.7	36.4 ± 0.8	35.9 ± 0.8	36.1 ± 0.6	35.1 ± 1.3	37.4 ± 1.0

One-way ANOVA followed by Tukey's post hoc test was used for statistical comparisons (n = 3). Values are expressed as mean ± SEM. Different letters within each row indicate statistically significant differences among NMN treatments at $p < 0.05$. Abbreviations are as defined in Table S1. a,b,c,d Different letters indicate significant differences between mean values for a given behavior ($p < 0.05$).

2. Supplementary Figures

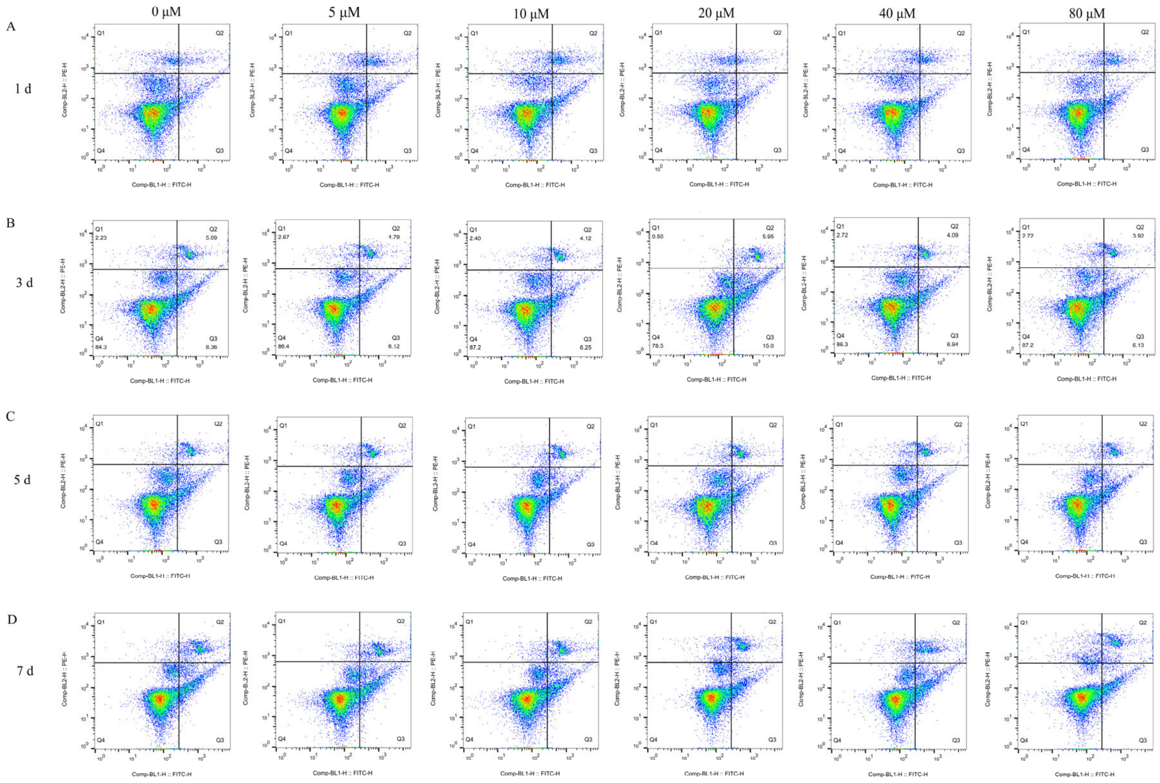

Figure S1. Flow cytometry analysis of the effect of NMN supplementation in extender on boar sperm viability and acrosome integrity. (A) Boar sperm viability and acrosome integrity on 1 days; (B) Boar sperm viability and acrosome integrity on 3 days; (C) Boar sperm viability and acrosome integrity on 5 days; (D) Boar sperm viability and acrosome integrity on 7 days.

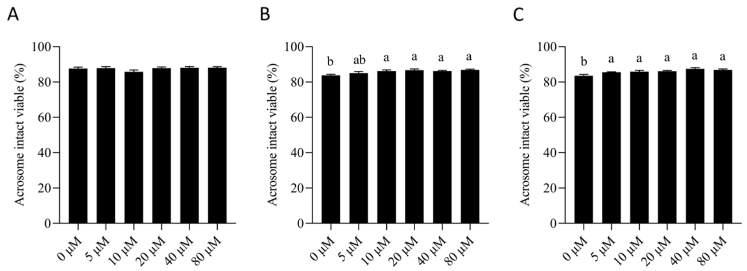

Figure S2. NMN supplementation maintains boar sperm viability and acrosome integrity during liquid storage. (A–C) Representative flow cytometry dot plots of viable sperm with intact acrosomes after 1, 3, and 5 days of storage. Values are expressed as mean ± SEM (n = 3). Different letters represent significant differences between treatments ($p < 0.05$).
